# Supplementary material for: The causal relationship between the human gut microbiota and pyogenic arthritis: a Mendelian randomization study
Source: Front Cell Infect Microbiol. 2024 Nov 26;14:1452480. doi: 10.3389/fcimb.2024.1452480 (PMC11629706; doi:10.3389/fcimb.2024.1452480)
Supplement: Supplementary file 1 [file DataSheet1.zip › mendelian test/ebi-a-GCST90027753.csv_forest.pdf]

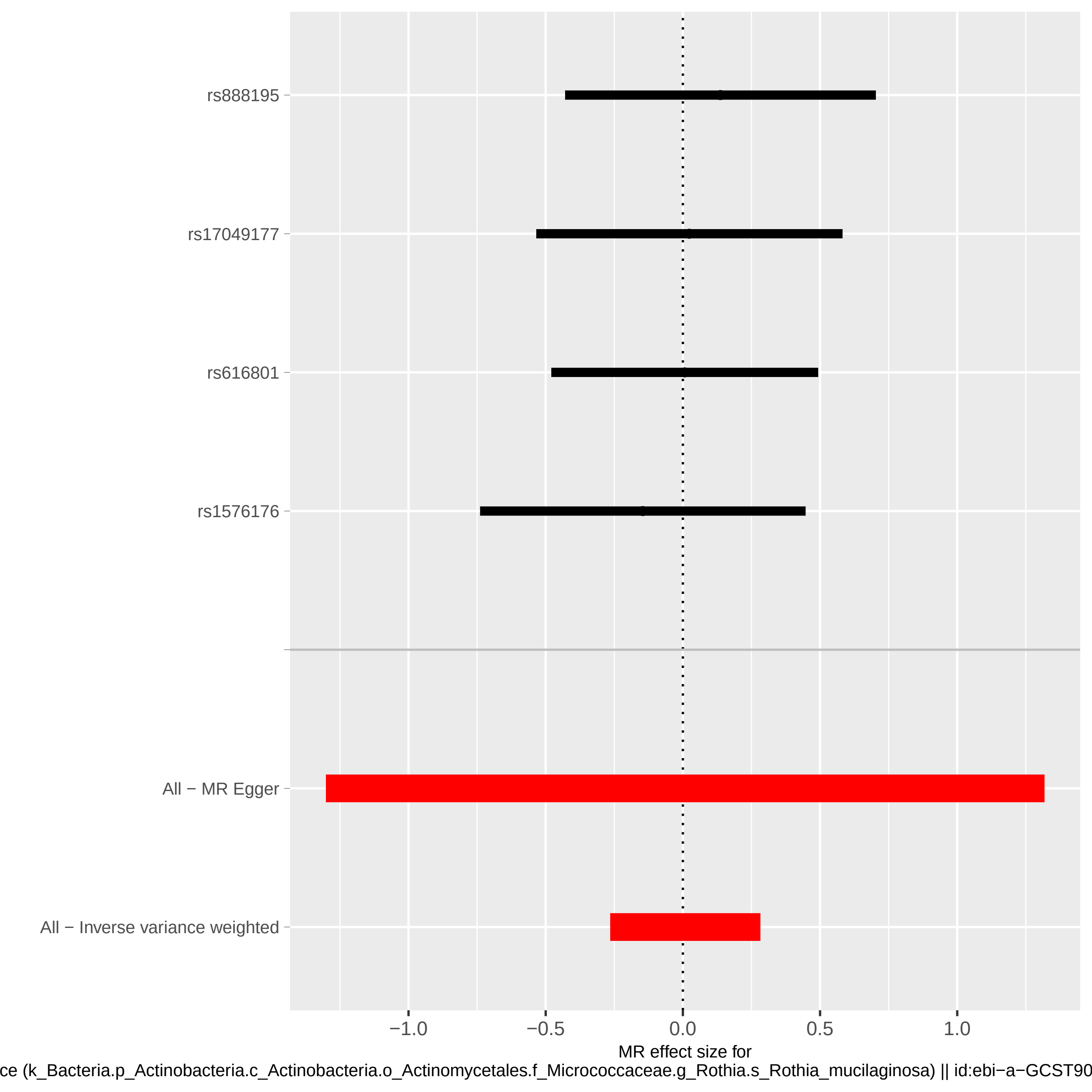

rs888195
rs17049177
rs616801
rs1576176
All − MR Egger
All − Inverse variance weighted
−1.0
−0.5
0.0
0.5
1.0
MR effect size for
ce (k_Bacteria.p_Actinobacteria.c_Actinobacteria.o_Actinomycetales.f_Micrococcaceae.g_Rothia.s_Rothia_mucilaginosa) || id:ebi−a−GCST90
